# Seedless formation of a landrace ‘Sanenashi’ (*Pyrus* L.) collected from Northern Tohoku, Japan

**DOI:** 10.1270/jsbbs.24003

**Published:** 2024-11-27

**Authors:** Shohei Mitsuhashi, Seiji Nakano, Chiyomi Uematsu, Hironori Katayama

**Affiliations:** 1 Food Resources Education and Research Center, Faculty of Agriculture, Kobe University, Kasai, Hyogo 675-2103, Japan; 3 Botanical Gardens, Graduate School of Science, Osaka City University, Katano, Osaka 576-0004, Japan

**Keywords:** pseudo-parthenocarpy, *Pyrus ussuriensis* var. *aromatica*, Sanenashi, seedless pear

## Abstract

‘Sanenashi’ is a landrace of *Pyrus ussuriensis* var. *aromatica* (Iwateyamanashi) with seedless fruit originating from northern Tohoku, Japan. To determine the mechanism of seedless fruit formation, we compared the number of styles in the pistil, fruit, seed set and the pollen tube growth between ‘Sanenashi’ and the Japanese pear cultivar ‘Kosui’. Morphological variations such as short or browning pistils were observed in 64.2% of ‘Sanenashi’ and 5.9% of ‘Kosui’. The initial fruit set rate of ‘Sanenashi’ was 48.3% at 4 weeks after cross-pollination with pear, and there were no fruit sets with peach pollen and non-pollinated sections at 8 weeks. Although the seed sets of ‘Sanenashi’ fruit were much lower than that of ‘Kosui’, 55.3% of viable ‘Sanenashi’ seeds germinated. Pollen tube growths were observed in the stigma and style of ‘Sanenashi’, but whether they reached the ovary could not be confirmed. Single sequence repeat (SSR) alleles of F_1_ progenies between ‘Sanenashi’ and pear cultivars were presented by five SSR markers. These results suggest that the seedless fruit formation in ‘Sanenashi’ corresponds with pseudo-parthenocarpy (stenospermocarpy) because pollination by pear pollen is necessary for the fruit set. However, the results do not fully confirm this hypothesis and require further experiments.

## Introduction

Consumers tend to prefer seedless fruit because it is easier to consume than seeded fruit. Therefore, the mutations in naturally occurring seedless fruit have been selected and maintained by traditional methods of propagation, such as grafting. Seedless watermelons and grapes have been established by artificial breeding (Andrus *et al.* 1971, Aradhya *et al.* 2003). Many studies have investigated the mechanisms of seedless fruit formation (e.g., in citrus and grape). Parthenocarpy is a common mechanism of seedless fruit formation that does not require pollination and fertilization (Nitsch 1965). However, Winkler (1908) has identified other mechanisms requiring pollination or other stimulation, namely as heat and hormone stresses. Autonomic-parthenocarpy, not requiring pollination, occurs in various Japanese persimmon cultivars (e.g., ‘Hiratanenashi’ and ‘Miyazaki-mukaku’) and the ‘Black Collins’ grape, and stimulative-parthenocarpy occurs in the citrus ‘Hyuuga-natsu’ and European pear ‘Seckel’. Kobel (1931) has identified another pollination-based mechanism, namely, pseudo-parthenocarpy. In parthenocarpic fruit, the ovules are unfertilized, and in pseudo-parthenocarpic fruit, the ovules can be normally fertilized, after which they show a sudden arrest of growth and disappear or form rudimentary structures. This phenomenon is observed in certain varieties of European pears, cherry, persimmon, grape, avocado, and cucumber.

In pears (*Pyrus* spp.), although seedless European and Chinese cultivars have been established, no seedless Japanese cultivar exists, except for hybrids between European and Japanese pear cultivars (Moriya *et al.* 2005, Nishitani *et al.* 2012, Nyeki *et al.* 1998). Katayama and Uematsu (2006) reported that ‘Sanenashi’ (Iwate-mukaku), a landrace of *Pyrus ussuriensis* var. *aromatica* (Iwateyamanashi) collected from northern Tohoku, Japan, was favored during the Edo era (ca. 400 years ago) because of its good taste, aroma, and small seedless core; thus it was widely disseminated by grafting. Although the aroma of ‘Sanenashi’ has been evaluated using sensory and chemical techniques (Katayama *et al.* 2013), the mechanism of seedless fruit formation remains largely unclear.

Adachi (1935) has reported that the mechanism of seedless fruit formation in ‘Mukaku-nashi’ (synonymous with ‘Sanenashi’) is caused by differences in the flower organ that directly affect female sterility, such as incomplete stigma and few pistils. Accordingly, this study investigated the mechanism of seedless fruit formation in ‘Sanenashi’ by comparing the rates of seedlessness and germination, as well as the histological pollen tube growth between ‘Sanenashi’ and a Japanese pear cultivar, ‘Kosui’.

## Materials and Methods

### Plant materials

The seedless pear landrace ‘Sanenashi’, two Japanese pear cultivars (‘Kosui’ and ‘Chojuro’), and the peach cultivar ‘Nagasawa Hakuho’, were used in this study. The plants were maintained at Food Resources Education and Research Center, Kobe University.

### Morphological variations in pistil and pollination experiments

Field experiments were conducted for 2 years: 2010 and 2011. Morphological changes in the pistils of ‘Sanenashi’ and ‘Kosui’ were observed in 2010. The number of pistils per flower was calculated and the styles were categorized according to their shape and color as follows: “normal”, “flat stigma”, “dried stigma”, “short pistil” or “browning”. Using one or two trees for each pear, we conducted cross-pollinations by collecting anthers from pollen parents selected from the second to fourth flowers in a nonadjacent flower cluster. Flowers were emasculated during anthesis by using tweezers. Cross- and non-pollination were performed on April 17, 2010, and April 16, 2011. ‘Sanenashi’ was pollinated with pollen from ‘Kosui’, ‘Chojuro’, and ‘Nagasawa Hakuho’ (peach) for 2 years and covered with paraffin paper bags to avoid pollen contamination. Fruit sets were observed from 1 to 8 weeks after pollination. Cross-pollinated fruits from ‘Sanenashi’ and ‘Kosui’ were harvested at 16 weeks, and the number of seeds per locule was counted. The diameter and major axis of ‘Sanenashi’ seeds were measured using an electric slide caliper. Seeds were categorized by size (Student’s *t* test, *p* < 0.01) and appearance as “mature” (viable), “immature” (inviable black seed), and “aborted” (white seed). ‘Kosui’ seeds were categorized “mature” and “immature” based on appearance because clear differences were observed.

### Pollen tube growth

We examined pollen grain germination and tube growth as described by Sitch and Snape (1987). Briefly, in 2010, pistils were fixed 48 h after pollination, initially washed in water, hydrolyzed in 70% lactic acid in a boiling water bath, and then cooled. The softened pistils were washed in distilled water and immersed in 0.1 M K_3_PO_4_ for 16 h. The pistils were stained with Aniline blue [0.2% (w/v) in 0.1 M K_3_HPO_4_]. Each pistil was dissected using a fluorescence microscope (HB-10101AF, Nikon, Tokyo, Japan) to expose the ovule.

### Seed germination rates and hybrid identification

After breaking dormancy in 2010, seeds were planted in a plug tray and maintained in a growth chamber. The number and rate of seed germination were calculated. For determining the fertilization status of germinated progenies, F_1_ hybrids were identified with five single sequence repeat (SSR) markers (NH011b, NB104a, NH009b, NH025a, and NH201a; Table 1) as described by Yamamoto *et al.* (2002a, 2002b). Total DNAs of the F_1_ hybrids were extracted as described by Sassa (2007). Polymerase chain Reactions (PCR) were performed using the total DNAs under the following conditions: 94°C for 2 m, 30 cycles of (94°C for 1 m, 55°C for 1 m, 72°C for 1 m), and 72°C for 2 m. PCR amplification was conducted using a GeneAmp PCR system 9700 (Thermo Fisher Scientific, Waltham, MA, USA) and analyzed using an Applied Biosystems 3500 genetic analyzer with GeneScan software (Thermo Fisher Scientific). Internal standard DNA (GeneScan 350 TAMRA, Thermo Fisher Scientific) was used to calculate the amplified fragment size.

## Results

### Variations in pistil morphology

**Fig. 1.**
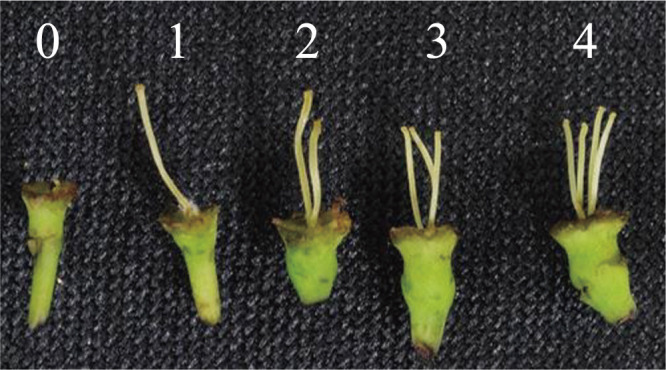
Morphological variation in pistils of ‘Sanenashi’. The numbers indicate the number of pistils.

No notable weather anomalies and no frost damage were observed during the experimental period. The mean numbers of pistils in flowers varied: The highest mean rates of pistils per flower in ‘Sanenashi’ were 48.9% for two pistils and 32.6% for three pistils, and the highest mean rates of pistils per flower in ‘Kosui’ were 37.7% and 26.5% for seven and eight pistils during the 2 years (Fig. 1, Table 2).

**Fig. 2.**
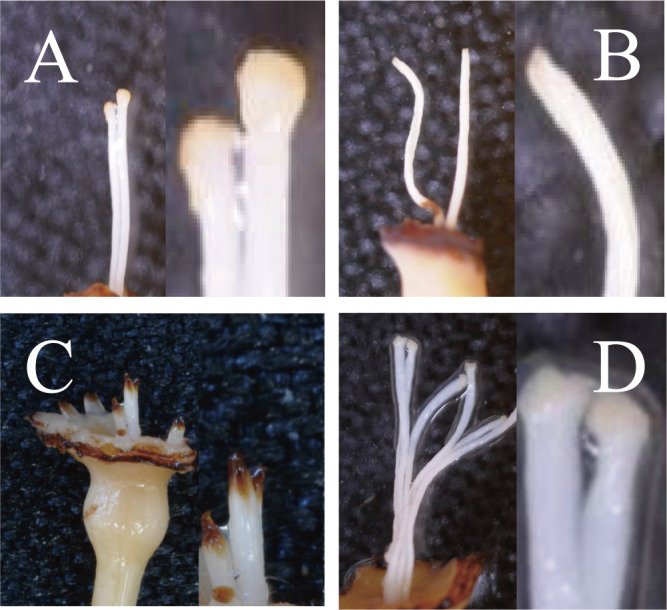
Morphological variations in the pistils and stigmas of ‘Sanenashi’. (A) Normal pistil and stigma. (B) Flat and dried stigma. (C) Short and browning pistil. (D) Normal pistil in ‘Kosui’.

Morphological changes were observed in 374 ‘Sanenashi’ pistils from 2010, with a 64.2% appearance rate of abnormalities within the pistil and stigma. The various shapes were categorized into three types: 189 pistils (50.5%) with a flat stigma, 2 pistils (0.5%) with a dried stigma, and 49 pistils (13.1%) that were short and/or browning (Fig. 2, Table 3). From the 205 pistils in ‘Kosui’, 12 (5.9%) were short and/or browning.

In one flower, there were some with the same shape of pistil and others with different shapes of pistils. In ‘Sanenashi’, there were 27 (17.2%) flowers with only normal pistils, 78 (49.7%) flowers with only abnormal pistils, and 52 (33.1%) flowers with mixed (normal and abnormal), respectively. In ‘Kosui’, there were no flowers with only abnormal pistils (Table 4). These findings indicate that ‘Sanenashi’ had more morphological variations in the pistil and stigma than ‘Kosui’.

### Fruit and seed sets

The fruit sets were counted in ‘Sanenashi and ‘Kosui’ at six time points (0, 1, 2, 3, 4, and 8 weeks) over 2 years. In ‘Sanenashi’, a total of 690 flowers were cross-pollinated with ‘Chojuro’ and ‘Kosui’, of which >40% produced fruit sets after 8 weeks. No fruit sets were observed in non-pollinated ‘Sanenashi’ at 8 weeks. Moreover, no fruit sets were observed in the ‘Nagasawa Hakuho’ (peach) pollen experiment at 4 weeks. In ‘Kosui’, 76 flowers were pollinated with ‘Chojuro’ and 28 flowers with peach for 2 years. The mean fruit set rates of ‘Kosui’ cross-pollinated with ‘Chojuro’ and peach pollens were 56.6% and 3.6%, respectively, at 8 weeks. The mean fruit set rate in non-pollinated ‘Kosui’ was 8.3% at 8 weeks (Table 5).

**Fig. 3.**
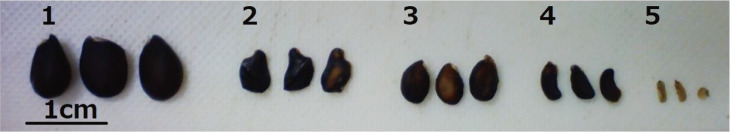
Mature and immature seeds of ‘Kosui’ and ‘Sanenashi’. 1, mature seeds in ‘Kosui’; 2, immature seeds in ‘Kosui’; 3, mature seeds in ‘Sanenashi’; 4, immature seeds in ‘Sanenashi’; 5, aborted seeds in ‘Sanenashi’.

The mean locule number per fruit in ‘Sanenashi’ pollinated with ‘Kosui’ and ‘Chojuro’ ranged from 2.3 to 2.6 over the 2 years. In ‘Kosui’, the number of cross-pollinated locule was 7.0–8.0 (Table 6). The mean diameters (lengths) of mature seeds in ‘Sanenashi’ pollinated with ‘Kosui’ and ‘Chojuro’ were 2.1 (4.9) mm and 2.8 (5.0) mm, those of immature seeds were 0.9 (3.7) mm and 1.2 (3.7) mm, and those of aborted seeds were 0.7 (2.5) mm and 0.7 (2.5) mm, respectively. Seed size and lengths varied significantly between categories (Student’s *t* test, *p* < 0.01; Fig. 3, Table 7).

The rates of immature and aborted seeds were 96.5% and 92.1% for in ‘Sanenashi’ pollinated with ‘Kosui’ and 85.4% and 88.5% for in ‘Sanenashi’ pollinated with ‘Chojuro’, respectively. Non-pollinated and cross-pollinated ‘Sanenashi’ seeds by peach pollens were not observed. The seeds of ‘Kosui’ pollinated with ‘Chojuro’ were categorized into mature (viable) and immature (inviable). The rates of mature seeds in ‘Kosui’ pollinated with ‘Chojuro’ were 22.9% and 52.5%, respectively. Thus, the rate of mature seeds was considerably lower in ‘Sanenashi’ than ‘Kosui’. In 2010, 9.5% of non-pollinated ‘Kosui’ seeds were mature (Table 6).

After seed dormancy, the germination rates of 10 and 16 mature seeds from ‘Sanenashi’ pollinated by ‘Kosui’ or ‘Chojuro’ pollen were 43.5% and 72.7%, respectively, for 2 years. The germination rate of mature ‘Kosui’ seeds was relatively higher than that in ‘Sanenashi’ (Table 8).

### Pollen tube growth

**Fig. 4.**
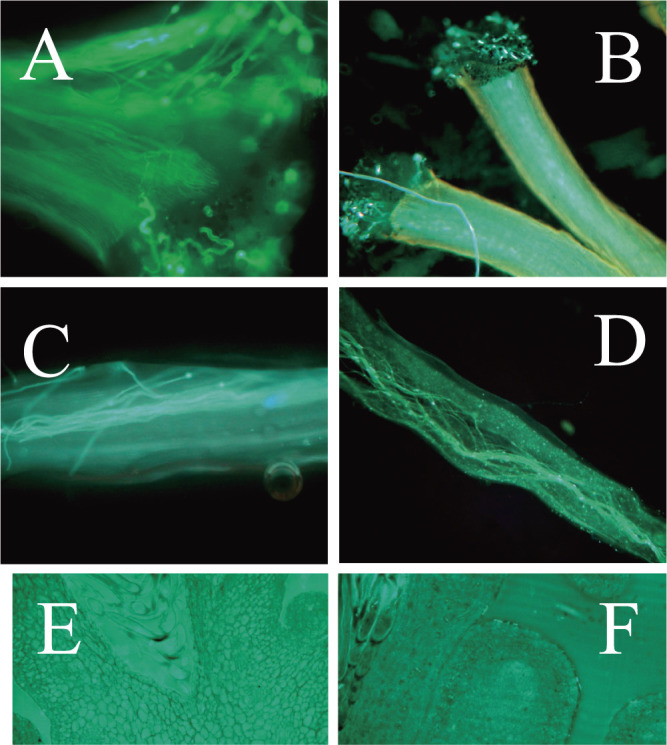
Fluorescence microscopy images of pollen tube growth in pistils of ‘Sanenashi’ and ‘Kosui’. Several pollen tubes can be seen growing in the stigma of ‘Sanenashi’ (A), stigma of ‘Kosui’ (B), transmitting tissue of ‘Sanenashi’ (C), transmitting tissue of ‘Kosui’ (D), around ovule in ‘Sanenashi’ (E), and around ovule in ‘Kosui’ (F).

We further investigated seed formation in ‘Sanenashi’ based on pollen tube growth in the pistils of both soft and resin-embedded tissue. Pollen germination was observed in the pistils of ‘Sanenashi’ pollinated by ‘Chojuro’ and ‘Kosui’ pollen. We observed callose plugs in the stigma, the tip and base of the style in ‘Sanenashi’, consistent with the observations for ‘Kosui’ pollinated with ‘Chojuro’. The stigma and style of ‘Kosui’ had more pollen tubes than those of ‘Sanenashi’. Although a few pollen tubes occurred near the ovule in both ‘Sanenashi’ and ‘Kosui’, no pollen tubes were observed to reach the embryo sac in either soft or resin-embedded tissue sections (Fig. 4).

### Identification of F_1_ genotype using SSR markers

We screened five SSR markers (derived from European pear and Japanese pear varieties) in 14 F_1_ seedlings (13 mature and 1 immature seeds) of ‘Sanenashi’ pollinated with a ‘Kosui’ or ‘Chojuro’ pollen (Sawamura *et al.* 2004, Yamamoto *et al.* 2002a, 2002b, Table 1). The F_1_-progeny samples were compared with control ‘Sanenashi’, ‘Kosui’, and ‘Chojuro’ samples. Three alleles in the F_1_ progeny were discriminated by NB104a, NH009b, and NH201a; four and six alleles were detected with NH011b and NH025a (Table 9). These results confirmed that all F_1_ progeny were hybrids of ‘Sanenashi’ and ‘Kosui’ or ‘Chojuro’.

## Discussion

### Characterization of flower organ in ‘Sanenashi’

‘Sanenashi’ and ‘Kosui’ pistils had many morphological differences (Tables 2–4). The number of pistils per flower was mainly two or three in ‘Sanenashi’ and in contrast with five or more in ‘Kosui’ or the typical *P. ussuriensis* and *P. pyrifolia*. Morphological variations such as short or browning pistils were observed in 64.2% of ‘Sanenashi’ and 5.9% of ‘Kosui’. The initial fruit set rates in ‘Sanenashi’ were 48.3% and 0.2%, and those of ‘Kosui’ were 76.3% and 32.0%, 4 weeks after cross- and non-pollination, respectively (Table 5). Thus, morphological differences in ‘Sanenashi’ pistils can somehow prevent their pollination or fertilization. For example, secretion around the stigmas can influence pollen germination (Edlund *et al.* 2004).

Chauhan *et al.* (1987) reported morphological differences in *Crescentia cujete* L., where the stigma surfaces of both seeded and seedless plants exhibited distinct characteristics under an electron microscope; the former showed small and loosely arranged wet papillae, and the latter displayed large compactly arranged dry papillae.

In ‘Sanenashi’, most pistils with morphological variation had small or absent stigma (Fig. 2). Adachi (1935) observed in ‘Sanenashi’ approximately 1–2 normal stigma per flower, with the majority abnormal, which may lead to sterility. Thus, we hypothesized that pollen attachment is poorer in abnormal (e.g., dry or shape of stigma surface) than normal stigmas. In this study, ‘Sanenashi’ had a mixture of flowers, some with the same type of pistil and others with different types of pistils (Table 4). It is challenging to trace the association between morphological characteristics and seedless formation. However, we may comprehend it using the flower with all the same type of pistils. Further research should consider the association between morphological differences and fertilization, such as pollen germination, growth, and fecundation in affected pistils.

### Parthenocarpy and pseudo-parthenocarpy

Approximately half of all ‘Sanenashi’ fruit were pollinated and set, whereas no fruits were non- or peach-pollinated and set (Table 5). These findings suggest that seedlessness in ‘Sanenashi’ is neither stimulative- nor autonomic-parthenocarpic. Therefore, further research should investigate the potential for pseudo-parthenocarpy to produce these phenotypes. By contrast, a few ‘Kosui’ non- and peach-pollinated fruits set, with most having only immature seeds (Tables 5, 6). This phenomenon is consistent with autonomic-parthenocarpy and that some *P. pyrifolia* cultivars, such as ‘Hosui’, show weak parthenocarpy but not seedlessness (Nishitani *et al.* 2012). However, the ‘Kosui’ non-pollinated section in 2010 obtained four mature seeds, despite non-pollinated pears with weak parthenocarpy being known to be unable to produce mature seeds. The cause of this phenomenon is unknown, but it might be due to a technical oversight in 2010, such as contamination due to incomplete bagging or the failure to emasculate the flowers, because no mature seeds were within the same section in 2011.

### Seed fertility

In ‘Sanenashi’, the total number of seeds was 520: 48 (9.2%) were mature, and 472 (90.8%) were abnormal (Table 6). Over 50% of the mature seeds germinated, and one of the abnormal seeds germinated (Table 8).

Pearson (1932, 1933) has reported that ‘Thompson Seedless’, a pseudo-parthenocarpic grape variety, ordinarily has four ovules in the ovary, of which at least one fertilizes and germinates, and the others are incomplete and degenerate without fertilization. The germinated ovules variously grow into aborted, imperfect, or perfect seeds (mostly aborted) that have previously been cultivated into embryo plants. Stout (1936) has found that due to a mixture of post-fertilization degeneration and non-fertilization phenomena, seedless fruit formation in ‘Thompson Seedless’ does not correspond completely to conventional pseudo-parthenocarpy and defines the mechanism as “stenospermocarpy”.

Because most of the seeds were aborted and imperfect, we hypothesize that seedlessness in ‘Sanenashi’ occurs by pseudo-parthenocarpy and that the characteristics of this mechanism may be similar to those of stenospermocarpy.

### Ovule fertilization and potential mechanism of seedless fruit formation

In ‘Sanenashi’ pistils, we observed pollen germination at the stigma along with pollen tubes growing through the stigma, tip of the style, and base of the style in both ‘Chojuro’ and ‘Kosui’ pollinated sections. However, fewer pollen tube growths were observed in ‘Sanenashi’ than in ‘Kosui’. We did not observe any germinated ovules. If seedless fruit formation in ‘Sanenashi’ is caused by pseudo-parthenocarpy, then ovules would be expected to germinate normally, after which most degenerate and are aborted. However, in this study, the pollen tubes were not observed to arrive at the ovules, which is inconsistent with pseudo-parthenocarpy and somewhat consistent with stenospermocarpy of ‘Thompson Seedless’, as described in the previous paragraph “Seed fertility”. These results also suggested that ‘Sanenashi’ could fruit by physical stimulation through pollination alone, but we rejected stimulative-parthenocarpy based on the peach pollination test. Notably, we did not observe the pollen tubes reaching the ovules in ‘Kosui’ control plants either, which may be due to inappropriate handling in the experiment or limitations in the experimental setup. Under the conditions of 48 h after pollination, the same as in this study, An *et al.* (2016) reported that many pollen tubes had reached the ovules, and Claessen *et al.* (2022) indicated that from 61.4% (*N* = 44) to 78.1% (*N* = 32) of the individuals had one or more pollen tubes visible at the base of the style. However, some studies observed the vicinity of the ovules at, for example, 72 h and 5 d after pollination (Sanzol and Herrero 2007, Yamashita *et al.* 1990). On the basis of our observations, we could not confirm that seedless fruit formation in ‘Sanenashi’ is pseudo-parthenocarpic (or stenospermocarpic), which might require further validation of the experimental system, namely modification of the observation time after pollination.

The SSR-marker analysis confirmed that the F_1_ progeny derived from the ‘Sanenashi’ mature or immature seeds had alleles from both ‘Sanenashi’ and ‘Kosui’ or ‘Chojuro’, suggesting hybridization with ‘Kosui’ or ‘Chojuro’ (Table 9). The results indicated that at least one ovule in ‘Sanenashi’ germinated, which is consistent with pseudo-parthenocarpy (stenospermocarpy).

In conclusion, seedless fruit formation in ‘Sanenashi’ likely occurs through a pseudo-parthenocarpic (stenospermocarpic) mechanism. However, this hypothesis is not fully confirmed by the results within this study and requires further experimental validation, for example, assessing the relationship between the morphological differences in pistils and fertilization rates, identifying stenospermocarpic degeneration of the ovules, or validating stimulative-parthenocarpy through pollination with sterile pollen.

## Author Contribution Statement

S.M., S.N, C.U. and H.K. conceived and designed the research. S.M. and S.N. conducted the experiments and prepared samples. S.M. and S.N. analyzed the data. C.U. and H.K. supervised the experiments. All authors contributed to the development of this manuscript.

## Figures and Tables

**Table 1.** SSR markers used in this study

SSR name	Label	Origin	Primer sequence (5ʹ-3ʹ)	Motif	Reference
NH011b	fluo (Vic)	‘Hosui’	F GGTTCACATAGAGAGAGAGAG R GTTTCTTTTTGCCGTTGGACCGAGC	(AG)9AA(AG)7	Yamamoto *et al.* 2002a and 2002b
NH009b	fluo (Fam)	‘Hosui’	F CCGAGCACTACCATTGA R GTTTCTTCGTCTGTTTACCGCTTCT	(AG)20	
NH025a	fluo (Hex)	‘Hosui’	F CTGGACACAAACATTCAAGAGGG R CACACCAGAAACTCCAAAACAGG	(AG)21(GA)4	
NB104a	fluo (Fam)	‘Bartlett’	F TCGGAGAGGAAGAGTTGGAGGA R AGGTCCGTGCCCAGTTTCTTTC	(GT)3GC(GT)5(GA)2TA(GA)5	Sawamura *et al.* 2004
NH201a	fluo (Fam)	‘Hosui’	F GTTTTGCTGCAATATCTCGCTA R GTTTCTTGATTGGAAGTGGATGGAGGA	(CA)14	

**Table 2.** Number and appearance ratio (%) of the pistil per flower in ‘Sanenashi’ and ‘Kosui’ for 2 years

Sample	Year	No. of flowers	No. of pistil																									
			0		1		2		3		4		5		6		7		8		9		10		11		12	
‘Sanenashi’	2011	1076	10	(0.9%)	110	(10.2%)	380	(35.3%)	414	(38.5%)	127	(11.8%)	35	(3.3%)	0	(0.0%)	0	(0.0%)	0	(0.0%)	0	(0.0%)	0	(0.0%)	0	(0.0%)	0	(0.0%)
	2010	1213	2	(0.2%)	90	(7.4%)	738	(60.8%)	331	(27.3%)	47	(3.9%)	5	(0.4%)	0	(0.0%)	0	(0.0%)	0	(0.0%)	0	(0.0%)	0	(0.0%)	0	(0.0%)	0	(0.0%)
	Mean	1144	6	(0.5%)	100	(8.7%)	559	(48.9%)	372.5	(32.6%)	87	(7.6%)	20	(1.7%)	0	(0.0%)	0	(0.0%)	0	(0.0%)	0	(0.0%)	0	(0.0%)	0	(0.0%)	0	(0.0%)
‘Kosui’	2011	285	0	(0.0%)	0	(0.0%)	0	(0.0%)	0	(0.0%)	2	(0.7%)	24	(8.4%)	40	(14.0%)	91	(31.9%)	93	(32.6%)	28	(9.8%)	6	(2.1%)	0	(0.0%)	1	(0.4%)
	2010	153	0	(0.0%)	0	(0.0%)	0	(0.0%)	0	(0.0%)	2	(1.3%)	15	(9.8%)	38	(24.8%)	74	(48.4%)	23	(15.0%)	0	(0.0%)	1	(0.7%)	0	(0.0%)	0	(0.0%)
	Mean	219	0	(0.0%)	0	(0.0%)	0	(0.0%)	0	(0.0%)	2	(0.9%)	19.5	(8.9%)	39	(17.8%)	82.5	(37.7%)	58	(26.5%)	14	(6.4%)	3.5	(1.6%)	0	(0.0%)	0.5	(0.2%)

**Table 3.** Number and appearance ratio (%) of morphological changes for stigma and pistil in ‘Sanenashi’ and ‘Kosui’ (2010)

Sample	No. of pistil	Normal		Abnormal		Abnormal stigma					Abnormal pistil	
						Flat		Dried			Short and/or Browning	
‘Sanenashi’	374	134	(35.8%)	240	(64.2%)	189	(50.5%)	2	(0.5%)		49	(13.1%)
‘Kosui’	205	193	(94.1%)	12	(5.9%)	0	(0.0%)	0	(0.0%)		12	(5.9%)

**Table 4.** Number and appearance ratio (%) of the pistil type per flower in ‘Sanenashi’ and ‘Kosui’ (2010)

Sample	No. of flower	Normal pistil		Abnormal pistil		Mixed (Normal and abnormal)	
‘Sanenashi’	157	27	(17.2%)	78	(49.7%)	52	(33.1%)
‘Kosui’	30	20	(66.7%)	0	(0.0%)	10	(33.3%)

**Table 5.** Number and appearance ratio (%) of fruit set by cross-pollination in ‘Sanenashi’ and ‘Kosui’ for 2 years

Sample	Pollen donor	Year	No. and rate of total fruit set											
			0 week		1 weeks		2 weeks		3 weeks		4 weeks		8 weeks	
‘Sanenashi’	‘Kosui’	2011	126	(100.0%)	125	(99.2%)	121	(96.0%)	76	(60.3%)	68	(54.0%)	51	(40.5%)
		2010	207	(100.0%)	175	(84.5%)	126	(60.9%)	106	(51.2%)	102	(49.3%)	92	(44.4%)
		Mean	166.5	(100.0%)	150	(90.1%)	123.5	(74.2%)	91	(54.7%)	85	(51.1%)	71.5	(42.9%)
	‘Chojuro’	2011	128	(100.0%)	127	(99.2%)	107	(83.6%)	60	(46.9%)	55	(43.0%)	53	(41.4%)
		2010	229	(100.0%)	220	(96.1%)	176	(76.9%)	111	(48.5%)	108	(47.2%)	92	(40.2%)
		Mean	178.5	(100.0%)	173.5	(97.2%)	141.5	(79.3%)	85.5	(47.9%)	81.5	(45.7%)	72.5	(40.6%)
	Pear cultivars		690	(100.0%)	647	(93.8%)	530	(76.8%)	353	(51.2%)	333	(48.3%)	288	(41.7%)
	Non-pollinated	2011	513	(100.0%)	512	(99.8%)	494	(96.3%)	13	(2.5%)	2	(0.4%)	0	(0.0%)
		2010	378	(100.0%)	346	(91.5%)	178	(47.1%)	2	(0.5%)	0	(0.0%)	0	(0.0%)
		Mean	445.5	(100.0%)	429	(96.3%)	336	(75.4%)	7.5	(1.7%)	1	(0.2%)	0	(0.0%)
	‘Nagasawa Hakuho’ (Peach)	2011	9	(100.0%)	9	(100.0%)	9	(100.0%)	1	(11.1%)	0	(0.0%)	0	(0.0%)
		2010	20	(100.0%)	20	(100.0%)	19	(95.0%)	2	(10.0%)	0	(0.0%)	0	(0.0%)
		Mean	14.5	(100.0%)	14.5	(100.0%)	14	(96.6%)	1.5	(10.3%)	0	(0.0%)	0	(0.0%)
	Others		920	(100.0%)	887	(96.4%)	700	(76.1%)	18	(2.0%)	2	(0.2%)	0	(0.0%)
‘Kosui’	‘Chojuro’	2011	29	(100.0%)	29	(100.0%)	29	(100.0%)	21	(72.4%)	19	(65.5%)	13	(44.8%)
		2010	47	(100.0%)	46	(97.9%)	46	(97.9%)	44	(93.6%)	39	(83.0%)	30	(63.8%)
		Mean	38	(100.0%)	37.5	(98.7%)	37.5	(98.7%)	32.5	(85.5%)	29	(76.3%)	21.5	(56.6%)
	Pear cultivars		76	(100.0%)	75	(98.7%)	75	(98.7%)	65	(85.5%)	58	(76.3%)	43	(56.6%)
	Non-pollinated	2011	121	(100.0%)	120	(99.2%)	115	(95.0%)	70	(57.9%)	47	(38.8%)	10	(8.3%)
		2010	48	(100.0%)	45	(93.8%)	10	(20.8%)	6	(12.5%)	4	(8.3%)	4	(8.3%)
		Mean	84.5	(100.0%)	82.5	(97.6%)	63	(74.6%)	38	(45.0%)	26	(30.8%)	7	(8.3%)
	‘Nagasawa Hakuho’ (Peach)	2011	22	(100.0%)	22	(100.0%)	22	(100.0%)	15	(68.2%)	9	(40.9%)	1	(4.5%)
		2010	6	(100.0%)	6	(100.0%)	6	(100.0%)	4	(66.7%)	3	(50.0%)	0	(0.0%)
		Mean	14	(100.0%)	14	(100.0%)	14	(100.0%)	9.5	(67.9%)	6	(42.9%)	0.5	(3.6%)
	Others		197	(100.0%)	193	(98.0%)	153	(77.7%)	95	(48.2%)	63	(32.0%)	15	(7.6%)

( ) denotes the appearance rate for the total number of flowers.

**Table 6.** Number and appearance ratio (%) of seed set in mature fruit in ‘Sanenashi’ and ‘Kosui’ for 2 years

Sample	Pollen donor	Year	Total no. of fruit	No. of locule/fruit *^a^*	Total no. of seeds	No. of seed observed in each category							
						Mature		Imperfect					
								Total		Immature		Aborted	
‘Sanenashi’	‘Kosui’	2011	26	2.5 ± 0.1	115	4	(3.5%)	111	(96.5%)	38	(33.0%)	73	(63.5%)
		2010	47	2.5 ± 0.1	202	16	(7.9%)	186	(92.1%)	30	(14.9%)	156	(77.2%)
	‘Chojuro’	2011	32	2.6 ± 0.1	151	22	(14.6%)	129	(85.4%)	67	(44.4%)	62	(41.1%)
		2010	13	2.3 ± 0.1	52	6	(11.5%)	46	(88.5%)	15	(28.8%)	31	(59.6%)
	Non-pollinated	2011	0	0.0 ± 0.0	0	0	(0.0%)	0	(0.0%)	0	(0.0%)	0	(0.0%)
		2010	0	0.0 ± 0.0	0	0	(0.0%)	0	(0.0%)	0	(0.0%)	0	(0.0%)
	‘Nagasawa Hakuho’ (Peach)	2011	0	0.0 ± 0.0	0	0	(0.0%)	0	(0.0%)	0	(0.0%)	0	(0.0%)
	Total		118	—	520	48	(9.2%)	472	(90.8%)	150	(28.8%)	322	(61.9%)
‘Kosui’	‘Chojuro’	2011	7	7.5 ± 0.2	105	24	(22.9%)	81	(77.1%)	81	(77.1%)	0	(0.0%)
		2010	22	7.0 ± 0.2	303	159	(52.5%)	144	(47.5%)	144	(47.5%)	0	(0.0%)
	Non-pollinated	2011	4	6.6 ± 0.9	56	0	(0.0%)	56	(100.0%)	56	(100.0%)	0	(0.0%)
		2010	4	7.0 ± 0.0	42	4	(9.5%)	38	(90.5%)	38	(90.5%)	0	(0.0%)
	‘Nagasawa Hakuho’ (Peach)	2011	1	8.0 ± 0.0	8	0	(0.0%)	8	(100.0%)	8	(100.0%)	0	(0.0%)
	Total		38	—	514	187	(36.4%)	327	(63.6%)	327	(63.6%)	0	0.0%

*^a^* Mean value ± standard error. Values in parentheses indicate the occurrence rate of total number of seeds. ( ) denotes the appearance rate for the total number of seeds.

**Table 7.** Mean seed diameter and length (major axis) in ‘Sanenashi’ by cross-pollination of ‘Kosui’ and ‘Chojuro’ for 2 years

Category	Year	Number of samples	Diameter (mm)*^a^* (Mean value ± standard error)	Length (mm)*^a^* (Mean value ± standard error)
Mature	2011	24	2.1 ± 0.1	4.9 ± 0.2
	2010	20	2.8 ± 0.1	5.0 ± 0.1
Immature	2011	93	0.9 ± 0.0	3.7 ± 0.1
	2010	20	1.2 ± 0.0	3.7 ± 0.1
Aborted	2011	57	0.7 ± 0.0	2.5 ± 0.1
	2010	20	0.7 ± 0.0	2.5 ± 0.1

*^a^* Three categorized values are significant at *p* < 0.01.

**Table 8.** Seed germination rates of F_1_ seedling obtained by cross-pollination for 2 years

Sample	Pollen donor	Year	Category	No. of seeds	No. of germinated seeds	Germination rate
‘Sanenashi’	‘Kosui’ ‘Chojuro’	2011	Mature	23	10	43.5%
			Immature	78	0	0.0%
			Aborted	40	0	0.0%
		2010	Mature	22	16	72.7%
			Immature	24	1	4.2%
			Aborted	20	0	0.0%
		Mean	Mature	23.5	13	55.3%
			Immature	51	0.5	1.0%
			Aborted	30	0	0.0%
‘Kosui’	‘Sanenashi’	2011	Mature	35	31	88.6%
			Immature	129	0	0.0%
			Aborted	0	0	0.0%
		2010	Mature	22	20	90.9%
			Immature	76	0	0.0%
			Aborted	0	0	0.0%
		Mean	Mature	28.5	25.5	89.5%
			Immature	102.5	0	0.0%
			Aborted	0	0	0.0%

**Table 9.** Genotypes of F_1_ progenies identified with 5 SSR markers

Sample*^a^*	SSR genotype (bp)				
	NH011b	NB104a	NH009b	NH025a	NH201a
S1	174/182	153/172	159/163	91/98	189/195
S2	170/184	153/172	149/163	68/101	189/195
S3	174/182	153/167	149/163	76/101	189/203
S4	170/184	153/172	149/163	98/101	189/195
S5	170/184	153/172	149/163	68/101	189/195
S6	170/184	153/172	149/163	68/101	189/195
S7	170/184	153/172	149/163	68/91	189/195
S8	170/184	153/172	149/163	68/101	189/195
S9	170/184	153/172	149/163	91/98	189/195
S10	170/184	153/172	149/163	68/91	189/195
S11	170/184	153/172	149/163	68/101	189/195
S12	170/184	153/172	149/163	98/101	189/195
S13	170/184	153/172	149/163	68/91	189/195
SIM1	170/184	153/172	149/163	68/91	189/195
‘Sanenashi’	170/174	153/172	149/159	91/101	189/189
‘Kosui’	182/184	172/172	163/163	68/98	195/195
‘Chojuro’	174/182	167/167	163/163	76/95	195/203

*^a^* S and SIM are derived from ‘Sanenashi’ mature seed and immature seed, respectively.
